# Experiences of postpartum Chinese women undergoing confinement practices: A qualitative meta‐synthesis

**DOI:** 10.1111/ijn.13251

**Published:** 2024-02-20

**Authors:** Jocelyn Khor Yun Xin, Goh Yong Shian, Tan Ting Han, Wilson Tam Wai San

**Affiliations:** ^1^ Alice Lee Centre for Nursing Studies, Yong Loo Lin School of Medicine National University of Singapore Singapore

**Keywords:** Chinese, confinement practices, ‘doing‐the‐month’, experience, meta‐synthesis, postpartum women

## Abstract

**Aims:**

We aim to review the experiences and needs of postpartum Chinese women undergoing ‘confinement’ or ‘doing‐the‐month’ a postpartum practice after childbirth.

**Methods:**

A meta‐synthesis was reported in alignment with the Enhancing Transparency in Reporting the Synthesis of Qualitative Research (ENTREQ) statement. Ten electronic databases were searched for eligible primary qualitative or mixed‐methods studies in English and Chinese from their inception until December 2021. Two reviewers independently reviewed the methodological quality of the included studies using the 10‐item Critical Appraisal Skills Program (CASP) qualitative checklist, with discrepancies resolved through discussions. The meta‐synthesis was conducted based on the two‐step approach by Sandelowski and Barroso.

**Results:**

Four themes were meta‐synthesized from 13 studies: ‘confinement’ as an essential practice; physical and psychological stressors during ‘confinement’; coping strategies by postpartum women; and needs for family, social, and professional support in enhancing satisfaction with ‘confinement’. The review showed that ‘confinement’ remains a significant practice shaped by socio‐cultural and health constructs.

**Conclusion:**

This review suggested re‐evaluating the ‘confinement’ practice and promoting evidence‐based care to improve maternal well‐being. Nurses and midwives should be cognisant of such postpartum customs and adopt non‐judgemental attitudes as early as the prenatal period to provide culturally sensitive care.

## Summary statement

What is already known about this topic?

- ‘Confinement’ is said to aid physical recovery after childbirth.
- Studies have shown that this practice can lead to the development of postnatal blues among some postpartum women.

What this paper adds?

- This review delineates how sociocultural factors govern postpartum women's beliefs, behaviours, and experiences during ‘confinement’, thus allowing healthcare professionals to better understand the influences of postpartum practices on maternal mental health.

The implications of this paper:

- The novel insights herein will aid healthcare professionals in formulating culturally appropriate care to improve postnatal confinement experiences and maternal psychological well‐being.

## 1 INTRODUCTION

Childbirth represents a biological phenomenon influenced by an individual's sociocultural beliefs and constructs. In particular, the postpartum period is shaped by such influences, especially for the practice of postnatal maternal confinement (Withers et al., 2018). In Chinese culture, ‘confinement’ or ‘doing‐the‐month’ is a practice lasting 30 to 40 days after childbirth, derived from the concept of *yin‐yang* harmony in traditional Chinese medicine. In this context, childbirth is perceived to upset the *yin‐yang* harmony, placing women in extreme *yin* associated with weakness and greater susceptibility to health problems (Lin et al., 2021). ‘Confinement’ is believed to restore the *yin‐yang* balance and improve health through adherence to dietary and behavioural rules, for example, staying indoors, eating *yang* food (e.g., ginger), and avoiding *yin* food (e.g., fruits) (see Table S1). During ‘confinement’, women are offered support in domestic and childcare responsibilities by caregivers, a role traditionally assumed by their mothers or mothers‐in‐law.

The postpartum period can be stressful for postpartum women, who undergo various physical, physiological, and psychological changes (Payne & Maguire, 2019). Besides the physical and physiological adaptations of their bodies, they are confronted by the challenging transition to motherhood. Furthermore, the rapid onset of these changes for the physically and emotionally vulnerable postpartum women takes a toll on their psychological well‐being, causing anxiety and depression in some women (Tsai & Wang, 2019). Notably, postpartum depression commonly sets in in the first month after childbirth (American Psychiatric Association, 2013).

Against this background, ‘confinement’ has been postulated as a beneficial practice against stressors after childbirth. It has been reported to improve postpartum women's psychological well‐being, contributing to a diminished risk of postpartum depression (Zheng et al., 2019). In particular, a lower risk of depressive symptoms was associated with a higher level of support received during ‘confinement’ (Yang et al., 2021), however, such views remain contradictory (Tsai & Wang, 2019). Moreover, in some studies, caregiver support has been identified as a risk factor for postpartum depression (Qi et al., 2021). Further compounding this was the inconclusive evidence on the protective effect of ‘confinement’ against postpartum depression (Yang et al., 2021).

The complex association between the ‘confinement’ practice, caregiver support, and maternal psychological well‐being remains poorly understood. While quantitative research cannot adequately delineate the role of ‘confinement’, qualitative research provides deeper insights into it. Some qualitative findings have suggested that the practice affected maternal psychological well‐being (Zheng et al., 2016, 2019), whereas others have reported that difficulties in following its restrictions and intergenerational conflicts with caregivers often precipitated distress among postpartum women (Peng et al., 2021). Given the conflicting qualitative findings on the role of ‘confinement’, it is necessary to consolidate the findings and better understand the interplay between social and cultural factors affecting maternal experiences and psychological well‐being.

Although existing reviews have explored the impacts of ‘confinement’ on various health outcomes including postpartum depression, they offered few meaningful insights into the experiences and needs (Liu et al., 2015; Wong & Fisher, 2009). Additionally, the focus of some literature has been overly broad: a meta‐ethnography on Southeast Asian and East Asian immigrant women's experiences of postpartum rituals included both Korean and Hmong practices and ‘confinement’ in their analysis (Vo & Desai, 2021). While the rituals might demonstrate similarities, intercultural differences would be present, due to which a culture‐specific discussion might be preferable to a broad and collective analysis. Moreover, most existing reviews on the experiences and needs of postpartum women undergoing ‘confinement’ have included studies in only English. The resultant omission of the copious evidence from Chinese publications might limit the comprehensiveness of their discussions and findings. Lastly, given the migration of Chinese communities in the context of globalization, healthcare professionals should take the initiative to understand ‘confinement’ in their delivery of culturally sensitive care.

Accordingly, the above discussion highlights a gap in the literature on the qualitative synthesis of the experiences and needs of postpartum women undergoing ‘confinement’. This meta‐synthesis therefore aimed to integrate existing qualitative evidence to answer the research question: ‘What are the experiences of postpartum women undergoing ‘confinement’? In this review, ‘confinement’ was defined as the Chinese postpartum practice consisting of dietary and behavioural prescriptions during the first postnatal month. By synthesizing available qualitative evidence on this practice, this meta‐synthesis was envisioned to enrich the existing literature and inform nurses on the provision of culturally appropriate care.

## 2 METHODS

### 2.1 Synthesis methodology

A meta‐synthesis was used to consolidate qualitative findings to both elicit and establish insightful meanings through an interpretative approach (Erwin et al., 2011). This meta‐synthesis was reported in alignment with the Enhancing Transparency in Reporting the Synthesis of Qualitative Research (ENTREQ) guidelines (Tong et al., 2012).

### 2.2 Approach to searching/data sources

An initial search was performed in PubMed with keywords such as ‘postpartum’, ‘postnatal’, ‘doing‐the‐month’, and ‘confinement’. To develop search strategies customized to each database, key terms and index terms were identified through textual analysis of titles and abstracts and combined with Boolean operators and truncation symbols (Table S2) A medical librarian was consulted to improve accuracy, and the search strategies were reviewed in accordance to the PRESS Guidelines (McGowan et al., 2016). Ten electronic databases (seven English and three Chinese) were included: PubMed, Embase, CINAHL, PsycINFO, Scopus, Web of Science, ProQuest Dissertations and Theses, China National Knowledge Infrastructure (CNKI), Airiti, and National Library of Theses and Dissertations (Taiwan). The search aimed at published and unpublished studies on ‘confinement’ on each database from its inception to December 2021. Moreover, the reference lists of the included studies and current reviews were manually searched to identify additional studies.

### 2.3 Inclusion criteria

Studies in English or Chinese were included if their *participants* were women aged ≥18 years who engaged in postpartum ‘confinement’. The *phenomenon of interest* revolved around their experiences and needs of ‘confinement’, with experiences being defined to incorporate perceptions, emotions, and challenges. No *context or geographical restriction* was imposed, given the widespread practice of ‘confinement’ across different settings. Our meta‐synthesis adopted a qualitative or mixed‐method *study design* (for studies under our examination that presented both qualitative and quantitative data, the qualitative data had to be distinguishable from the quantitative). The complete eligibility criteria are outlined in Table 1.

**TABLE 1:** Eligibility criteria.

Criteria	Inclusion	Exclusion
Population	‐ Women who have undergone ‘Tso‐Yueh‐Tzu’ during postpartum: ‐ Aged 18 years and above	‐ Suffered obstetric complications ‐ Suffered perinatal loss ‐ Pre‐existing psychological issues
Phenomenon of interest	‐ Experiences ‐ Needs ‐ Perceptions ‐ Emotions ‐ Attitude ‐ Challenges	‐ Narrative description of beliefs and rituals
Phenomenon of interest	‐ Tso‐Yueh‐Tzu	‐ Pregnancy ‐ Intrapartum
Context	‐ No restriction	
Type of design	‐ Primary qualitative studies (e.g., qualitative descriptive, phenomenology, grounded theory, feminist research, action research, case study, ethnography) ‐ Mixed method primary studies with distinguishable qualitative data from quantitative data	‐ Quantitative studies ‐ Quantitative surveys ‐ Exploratory studies ‐ Review
Year of publication	‐ No limit	
Publication type	‐ Published primary studies ‐ Unpublished thesis or dissertation	‐ Conference proceedings ‐ Abstracts only ‐ Books ‐ Review ‐ Editorials ‐ Missing full‐text
Language	‐ English ‐ Chinese	

### 2.4 Study screening methods

EndNote 20 (The EndNote Team, 2013) was used to collate all identified studies and remove duplicates. For the remaining studies, title‐ and abstract‐screening was performed independently by two reviewers (J.K.Y.X. and T.T.H.) to exclude irrelevant ones. Then, potentially eligible studies were retrieved for full‐text screening and assessed based on the eligibility criteria. Inter‐rater reliability (based on the Kappa statistic) was computed to determine the level of agreement between the reviewers. Discrepancies between them were resolved through discussion until consensus was reached.

**FIGURE 1:**
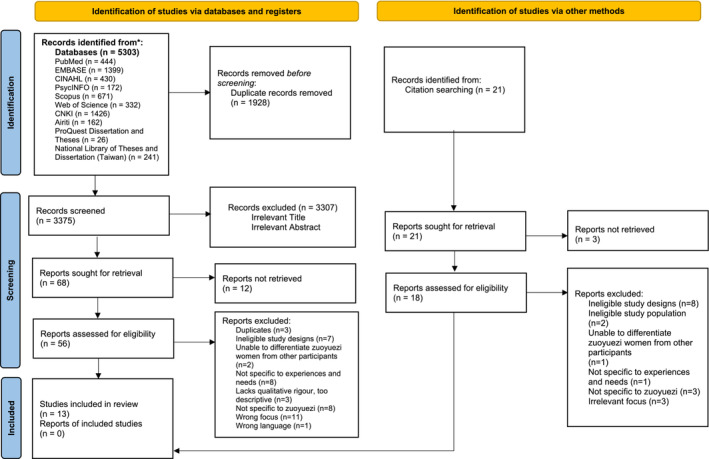
PRISMA 2020 flow diagram for new systematic reviews which included searches of databases, registers and other sources. *Consider, if feasible to do so, reporting the number of records identified from each database or register searched (rather than the total number across all databases/registers). **If automation tools were used, indicate how many records were excluded by a human and how many were excluded by automation tools. From*:* Page MJ, McKenzie JE, Bossuyt PM, Boutron I, Hoffmann TC, Mulrow CD, et al. the PRISMA 2020 statement: an updated guideline for reporting systematic reviews. BMJ 2021;372:n71. Doi: 10.1136/bmj.n71. For more information, visit: http://www.prisma‐statement.org/.

The literature search yielded 5324 studies, from among which 1949 duplicates were removed. The remaining 3375 studies were subjected to title‐ and abstract‐screening, of which 3307 were deemed irrelevant. Another 55 studies were excluded for not meeting the eligibility criteria. These led to the eventual inclusion of 13 studies in this meta‐synthesis. Substantial agreement between the reviewers was reflected by the kappa values of 0.73 for title‐ and abstract‐screening and 0.61 for full‐text screening. The PRISMA flow diagram (see Figure 1) outlines the screening.

### 2.5 Quality appraisal

To appraise the methodological rigour of the included studies, the 10‐item Critical Appraisal Skills Program (CASP) qualitative checklist was used (Critical Appraisal Skills Programme, 2018). The studies were appraised independently by two reviewers (J.K.Y.X. and T.T.H.), followed by a discussion to resolve discrepancies. For each assessed criterion, the studies were rated as Y for ‘Yes’, C for ‘Cannot tell’, or N for ‘No’. All studies were included regardless of their methodological quality, given the aim of quality appraisal to enhance the rigour of the synthesis, rather than to perform exclusionary filtering (Ludvigsen et al., 2016).

### 2.6 Data extraction

A pilot‐testing was conducted on the data‐extraction form (Table S3), by two reviewers (J.K.Y.X. and T.T.H.) before the form was used to elicit the relevant information, including authors, year of publication, study setting, aim, design, methodology (population characteristics and data collection), results (themes and subthemes), and conclusion. Verbatim and non‐verbatim statements encapsulating ‘confinement’ experiences and needs were also extracted. Discrepancies were discussed between the reviewers until a consensus was reached. A back and forward translation was performed by two reviewers (J.K.Y.X. and T.T.H.) as the findings in Chinese were translated into English for ease of data synthesis. For primary studies with missing information, their authors were contacted for clarification.

### 2.7 Data synthesis

Our data synthesis adopted the two‐step method by Sandelowski and Barroso (Ludvigsen et al., 2016; Sandelowski et al., 2007). First, it involved the meta‐summarizing (extraction, separation, grouping, and abstraction) of the findings into statement sets (Sandelowski et al., 2007). Then, in accordance to the thematic analysis by Braun and Clarke (Braun & Clarke, 2006), the findings were thematised via recurrent coding, reviewing, and theme‐seeking across the studies. Verbatim and non‐verbatim findings were subjected to line‐by‐line coding to create concepts, which were integrated to form descriptive themes and subthemes inductively and deductively. Subsequent studies were coded into existing concepts or consolidated to establish new concepts. In computing the effect size for each theme, the number of studies containing the theme was divided by the final number of studies, in order to measure how the corresponding data would support that theme (Onwuegbuzie, 2003). A meta‐synthesis then followed to integrate the meta‐summarized themes into more comprehensive and succinct themes, to provide insights into the experiences and needs of postpartum women undergoing ‘confinement’ (Sandelowski et al., 2007). Triangulation was maintained by comparing individual studies and re‐examining the themes to ensure congruence with the narratives across the studies. All the themes were discussed by both authors (J.K.Y.X. and T.T.H.) until a consensus was reached. Finally, the themes were organized and tabulated.

## 3 RESULTS

### 3.1 Study characteristics

Of the 13 studies under review (see Table 2), 11 were conducted in Asia (Hong Kong, *n* = 3; Taiwan, *n* = 6; and China, *n* = 2), while the rest were in North America (the United States of America, *n* = 1; and Canada, *n* = 1). An aggregate of 423 postpartum women, aged between 18 and 40, reported their experiences of ‘confinement’ in postpartum nursing‐care facilities and home‐based settings. They included both primiparas and multiparas of Chinese ethnicity, except for one study that recruited Vietnamese females who were married to Taiwanese males and had undergone ‘confinement’ in Taiwan (Lin et al., 2007). Methodologies varied across the studies: qualitative design (*n* = 4); qualitative descriptive design (*n* = 2); qualitative exploratory design (*n* = 3); ethnographic design (*n* = 1); grounded theory (*n* = 1); mixed‐methods design (*n* = 1); and descriptive method study (*n* = 1). Qualitative data were collected through semi‐structured or in‐depth interviews and focus‐group discussions. All but two studies reported their methods of data analysis: content analysis (*n* = 3); thematic analysis (*n* = 5); thematic and content analysis (*n* = 1); and Creswell's analysis (*n* = 2).

**TABLE 2:** Characteristics of included studies.

No.	Author (year), country	Aim of study	Methodology/data collection/analysis	Sampling method/sampling size/characteristics (age, ethnicity, parity, days of postpartum)	Themes/results (major)
1	Chang et al. (2018), Canada	To describe Chinese women's experiences with ‘zuo yue zi’ in British Columbia, Canada	Descriptive qualitative study/semistructured interviews via face‐to‐face and telephone/inductive content analysis	Purposive snowball sampling/*n* = 13/25–34 years old, Chinese, primiparas and multiparas, 4–6 weeks postpartum	Main theme: Chinese immigrant women's novel encounters with ‘zuo yue zi’. ‐ Expectations of ‘zuo yue zi’ ‐ Catalysts for ‘zuo yue zi’ ○ Chinese family members and friends as catalysts ○ Healthcare providers as catalysts ‐ Deterrents to ‘zuo yue zi’ ○ Chinese family members as deterrents ○ Unsatisfactory paid helpers as deterrents ○ Uninformed and insensitive healthcare providers as deterrents ‐ Mothers' modifications to ‘zuo yue zi’
2	Leung et al. (2005), Hong Kong	To examine the women's perceptions of stress and support in ‘doing the month’.	Qualitative study/face‐to‐face in‐depth interviews/content and thematic analysis	Purposive sampling/*n* = 20/18–35 years old, Chinese, primiparas and multiparas, 6 months postpartum	‐ Major care providers ‐ Types of support provided by care providers ‐ Sources of stress during the period of ‘doing the month’ ‐ Theme 1: Bound by the environmental constraints ‐ Theme 2: Difficulties in following the proscriptions of the ritual ‐ Theme 3: Conflicts between the parties involved ‐ Theme 4: The attainment of maternal role
3	Liu‐Chiang (1993), Taiwan	To explore the worries among Taiwanese mothers who participated in the Chinese ritual of Tso‐Yueh‐Tzu ritual in a center setting	Exploratory qualitative study/focus group discussions/qualitative content analysis	Purposive sampling/*n* = 21/20–35 years old, Chinese, primiparas, within 1 month postpartum	‐ Theme 1: Searching process to integrate the ‘self’ into the ritual of Tso‐Yueh‐Tzu ‐ Theme 2: Understanding that the newborn baby's care influences evaluation of the ‘self’ as a ‘good mother’ ‐ Theme 3: Decision‐making process that involved the ‘self’ needs to arrange the best baby care amidst being a ‘career‐woman’ ‐ Theme 4: Reconciling the need for ‘self’‐fulfilment with the demand to be a ‘family‐mother’.
4	Chen (2017), United States	To explore women's perception of zuoyuezi, and decision‐making process to do the month, as well as their takes on its constitutive Prescriptions and proscriptions	Qualitative study/semistructured interviews/not specified*	Not specified*/*n* = 27/Chinese, primiparas and multiparas, within 5 years postpartum	‐ The inevitability of zuoyuezi ‐ Negotiating the boundary of zuoyuezi knowledge ‐ Iced water at the hospital: When zuoyuezi meets biomedicine ‐ Family elders as filial authority ‐ Men's participation in zuoyuezi care ‐ Infant feeding during zuoyuezi ‐ Becoming a zuoyuezi care consumer ‐ Forging relations in paid postpartum care
5	Yeh et al. (2017), Taiwan	To explore new mothers' care needs from their own perspectives during the period of Doing the month.	Descriptive qualitative study/semistructured interviews/Creswell's data analysis	Purposive sampling/*n* = 27/25–39 years old, Chinese, primiparas, within 1 month postpartum	‐ Theme 1: The need to increase energy to gain more yang force ‐ Theme 2: The need to internalize mothering ‐ Theme 3: The need to be supported by the family and friends ‐ Theme 4: The need to be understood
6	Zheng et al. (2019), China	To explore Chinese primiparous women's experience of ‘doing the month’ and why Chinese women felt satisfied or dissatisfied with the experience.	Descriptive method study with qualitative data/open and closed‐ended surveys/thematic analysis	Purposive sampling, *n* = 420, 186 with qualitative results/20–40 years old, Chinese, primiparas, 6 weeks postpartum	‐ Why women felt satisfied with ‘doing the month’ 1. Support and rest 2. Sense of achievement 3. Positive thinking 4. Appreciative Attitudes ‐ Why women felt dissatisfied with ‘doing the month’ 1. Being tired of taboos 2. Conflicts with the Mother‐in‐law 3. Lack of family help And care 4. Undue expectations
7	Yeh et al. (2014), Taiwan	To explore how the traditional Chinese postpartum ritual of doing the month is being Reshaped for new mothers in a contemporary healthcare Context at a PNC in Taiwan.	Ethnographic study/semistructured interviews and key informant interviews/Creswell's data analysis	Not specified*/*n* = 27/26–38 years old, Chinese, primiparas within 4 weeks postpartum	‐ Reasons for performing doing the month at a postpartum nursing centre ‐ Dietary practices: Yang foods ‐ Dietary practices: Yin food ‐ Restricted physical activities ‐ Restricted hygiene practices ‐ Social proscriptions
8	Holroyd et al. (2013), Hong Kong	To examine how three generations of Chinese women practised ‘Doing the month’, specifically their behaviours and attitudes as well As to explore the influence of female family members on the practice.	Exploratory qualitative study/ethnographic interviews/thematic analysis	Convenience sampling/*n* = 42, 28 younger women, 14 older women/mean age: 29.5 years old, 56.2 years old, Chinese, primiparas, 6 weeks to 6th month postpartum	‐ Theme 1: Emotional status after childbirth ‐ Theme 2: Adopting specific customs for self‐protection ‐ Theme 3: Facing intergenerational difficulties and conflicts ‐ Theme 4: Source for ‘doing the month’ knowledge ‐ Theme 5: Gaining support ‐ Theme 6: Differences in ‘doing the month’ between the generations.
9	Holroyd et al. (2011), Hong Kong	To understand the female, family‐centred socializing experiences that shaped the practice of ‘doing the month’ in two generations of Hong Kong Chinese women.	Exploratory qualitative study/ethnographic semistructured interviews/thematic analysis	Convenience sampling/*n* = 32, 20 younger women, 12 older women/mean age: 29.5 years old, 56.0 years old, Chinese, primiparas, 6 weeks to 6th month postpartum	‐ Theme 1: Rites, rituals, and the establishment of a postpartum time period ‐ Theme 2: Finding ways to negotiate customary practices ‐ Theme 3: Burdensome nature of the practice ‐ Theme 4: Imperatives and punishments for breaking the practice
10	Sun (2015), China	To explore the postpartum experience of white‐collared women	Grounded theory/semistructured interviews/content analysis	Snowball sampling/*n* = 18/23–31 years old, Chinese, 2 to 10 months postpartum	‐ Self management ‐ Confinement diet ‐ Behavioural taboos and coping strategies ‐ Emotional status and regulation ‐ Body image management ‐ Recognition of the postpartum body ‐ Mother–infant relationship ‐ Relationship between mother‐in‐law and daughter‐in‐law ‐ Spousal relationship
11	Chiu (2012), Taiwan	To explore women's experience of ‘doing the month’ in modern society	Qualitative study/semistructured interviews and field observation/thematic analysis	Snowball sampling/*n* = 12/20–35 years old, Chinese, primiparas and multiparas, within 12 months postpartum	‐ Modern women's views and responses to confinement ‐ Behavioural taboos ‐ Dietary taboos ‐ Factors affecting women's choices of confinement ‐ Maternal mood towards postpartum physical changes ‐ Breastfeeding and the role of motherhood ‐ Spatial configuration and social relations
12	Lin et al. (2007), Taiwan	To explore the experience and feelings of Vietnamese primipara while ‘doing‐the‐month’ in Taiwan	Qualitative study/in‐depth interviews and field observation/thematic analysis	Purposive snowball sampling/*n* = 9/20–36 years old, Vietnamese, primiparas, not specified*	‐ Diet ‐ resistance vs acceptance under interaction of modernity and tradition ‐ Hygiene and cleanliness ‐ adherence vs autonomy under control of restrictions ‐ Home recuperation ‐ worry vs comfort in the limited space
13	Xu (2020), China	To explore the choices and pattern of confinement of women born after 1990s in terms of the clothing, food accommodation and activity and the factors influencing these choices	Mixed methods study/questionnaires, interview and field visits/not specified*	Purposive sampling/*n* = 400, 15 with qualitative results*/24–29 years old, Chinese, not specified*	‐ Clothing and supplies ‐ Diet ‐ Housing ventilation ‐ Hygiene practices ‐ Analysis of access to support ‐ Analysis of the husband's participation ‐ Influencing factors of the mode of confinement ○ Personal factors ○ Family factors ○ Family concept ○ Household income ○ Social factors ○ Socio‐cultural environment ○ Expectation of the role of a mother ‐ New consumerism culture

### 3.2 Quality appraisal of included studies

Thirteen studies were appraised (see Table S4), of which seven (Chang et al., 2018; Chiu, 2012; Leung et al., 2005; Liu‐Chiang, 1993; Sun, 2015; Yeh et al., 2014, 2017) met most of the items on the CASP checklist. Well‐addressed aspects included studies with clear and appropriate study aims, qualitative methodology, sampling strategies, and data collection, but also well‐considered ethical considerations and systematic reporting of findings and implications. Conversely, some inadequacies were noted. Most studies have not satisfactorily discussed the appropriateness of methodological designs, the rigour of data analysis, nor the researchers' reflexivity. Additionally, a mismatch was noted between the methods of data analysis used in some studies and the methods claimed by their authors.

### 3.3 Themes identified

Fourteen themes were generated from the meta‐summary of first‐ and second‐order constructs across the 13 studies, with effect sizes ranging from 38.5% to 100% (see Table S5) (Onwuegbuzie, 2003). These themes were meta‐synthesized into four interrelated themes: ‘confinement’ as an essential practice; physical and psychological stressors during ‘confinement’; coping strategies by postpartum women; and needs for family, social, and professional support in enhancing satisfaction with ‘confinement’ (see Table S6).

#### 3.3.1 Theme 1: ‘confinement’ as an essential practice

Ten studies reflected the postpartum women's perception of ‘confinement’ as an essential practice. Its significance stemmed from deep‐rooted socio‐cultural indoctrination that regarded it as ‘*a custom*’ ‘*passed down from generation to generation*’ (Chang et al., 2018; Chen, 2017; Chiu, 2012; Holroyd et al., 2011, 2013; Leung et al., 2005; Lin et al., 2007; Liu‐Chiang, 1993; Sun, 2015; Xu, 2020; Yeh et al., 2014; Zheng et al., 2019). Immersed in a cultural milieu where ‘confinement’ was practised by most, Chinese women grew up ‘*being told so often about its importance*’ by propagators, including their elders and friends (Chang et al., 2018; Chen, 2017; Chiu, 2012). The older generation were an especially potent factor in shaping this perception, as reflected by how the postpartum women perceived ‘*no harm*’ in complying with their advice, or by how they feared performing ‘confinement’ poorly and thus suffered in the manner described by them (Chang et al., 2018; Chen, 2017; Chiu, 2012; Holroyd et al., 2011, 2013; Leung et al., 2005; Sun, 2015; Xu, 2020).

The association between ‘confinement’ and health benefits also fostered its necessity. For example, the postpartum women perceived their bodies to be more vulnerable after childbirth and associated ‘confinement’ with various health advantages: regaining lost energy, increasing lactation, and promoting physiological and reproductive recovery (Chang et al., 2018; Chen, 2017; Chiu, 2012; Holroyd et al., 2011, 2013; Leung et al., 2005; Lin et al., 2007; Liu‐Chiang, 1993; Sun, 2015; Xu, 2020; Yeh et al., 2014, 2017; Zheng et al., 2019). Additionally, they worried about possible health repercussions from their negligence in performing ‘confinement’, risking ‘*back pain or headache at an older age if I don't do this and that right now* …’ (Chen, 2017; Holroyd et al., 2011; Liu‐Chiang, 1993). The postpartum women's ingrained belief in the significance of ‘confinement’ underlay their determination to continue practicing ‘confinement’.

#### 3.3.2 Theme 2: physical and psychological stressors during ‘confinement’

Eleven studies reported the postpartum women's negative feelings related to physical and psychological stressors during ‘confinement’. They were stressed, frustrated, and disgruntled with the need to adhere to burdensome restrictions, which inconveniently conflicted with their lifestyle: ‘*not washing hair in the first month after giving birth is unbearable*’ (Chen, 2017; Chiu, 2012; Holroyd et al., 2011, 2013; Leung et al., 2005; Lin et al., 2007; Sun, 2015; Xu, 2020; Yeh et al., 2014; Zheng et al., 2019). The multitude of behavioural rules of ‘confinement’ proved stifling to the postpartum women, during which the lack of freedom made some feel ‘*like being in jail*’ (Chiu, 2012; Holroyd et al., 2011; Leung et al., 2005; Lin et al., 2007; Sun, 2015). Moreover, concerns were raised with the burdensome costs incurred by ‘confinement’ (Chang et al., 2018; Chen, 2017;Chiu, 2012; Liu‐Chiang, 1993; Sun, 2015; Xu, 2020).

Despondency among the postpartum women also arose from juggling multiple responsibilities such as motherhood duties and infant care during ‘confinement’, precipitating an overwhelming sense of exhaustion (Chen, 2017; Chiu, 2012; Liu‐Chiang, 1993; Zheng et al., 2019). When confronted with challenges in breastfeeding and infant care, their stress and frustration amplified, leading to negative confinement experiences (Chen, 2017; Chiu, 2012; Liu‐Chiang, 1993; Sun, 2015; Xu, 2020; Yeh et al., 2017; Zheng et al., 2019). Primiparas, who sought to internalize their transition into motherhood through ‘confinement’, faced anxiety about the need to become conscientious mothers, compounded by their lack of confidence in infant care (Chiu, 2012; Holroyd et al., 2013; Leung et al., 2005; Liu‐Chiang, 1993; Xu, 2020; Yeh et al., 2017). The lack of guidance and support from care providers in overcoming these challenges further heightened their emotional toll: ‘*I called my mom, but she didn't know much either, and there were no nurses to help; I didn't know what to do at all. I was completely helpless*’ (Chiu, 2012). For those postpartum women in employment, work demands also created a sense of compulsion for them to end their confinement early to return to work (Chiu, 2012; Liu‐Chiang, 1993; Sun, 2015; Xu, 2020).

Inappropriate support from overzealous caregivers could compromise the postpartum women's maternal and self‐autonomy during ‘confinement’, as has been critiqued in 12 studies. When the caregivers overstepped and hindered mother‐infant bonding opportunities, frustration resulted among the postpartum women (Chen, 2017; Leung et al., 2005; Liu‐Chiang, 1993; Sun, 2015; Xu, 2020). The same applied to their loss of privacy when the caregivers trespassed on their personal spaces (Chiu, 2012; Leung et al., 2005; Sun, 2015). Furthermore, conflicts often emerged between the postpartum women and their family caregivers over differing opinions on ‘confinement’ practices, which added to their emotional stress (Chen, 2017; Chiu, 2012; Holroyd et al., 2011, 2013; Leung et al., 2005; Lin et al., 2007; Liu‐Chiang, 1993; Sun, 2015; Xu, 2020; Yeh et al., 2014; Zheng et al., 2019). Finally, when the caregivers imposed their opinions on the postpartum women, the threat to erode their autonomy strained the relationships: ‘*I told my mother‐in‐law, I didn't want to eat so much … but my mother‐in‐law continued*’ (Chang et al., 2018; Chen, 2017; Chiu, 2012; Leung et al., 2005; Liu‐Chiang, 1993; Sun, 2015; Xu, 2020). Against this background, these physical and psychological stressors collectively culminated in their negative ‘confinement’ experiences.

#### 3.3.3 Theme 3: coping strategies by postpartum women

Eleven studies discussed the postpartum women's strategies in coping with these stressors, which included accommodating themselves to the traditional ‘confinement’ customs or reshaping the customs to preserve self‐determination. Some handled the stressors by acquiescence under societal and cultural pressure (Chen, 2017; Chiu, 2012; Holroyd et al., 2011, 2013; Sun, 2015; Xu, 2020). They coped through self‐regulation strategies, including mood management and positive thinking, to internalize their negative emotions and perceptions. Others managed by reaching a compromise between themselves and their caregivers through negotiations to maintain harmony (Chen, 2017; Chiu, 2012; Holroyd et al., 2011, 2013; Sun, 2015; Xu, 2020; Zheng et al., 2019).

When the postpartum women could no longer tolerate it, they reshaped the traditional confinement customs to preserve self‐determination and fulfil their personal needs for comfort and maintenance of body figure: ‘*So I do what I feel like. Nothing is an absolute no‐no*’ (Chang et al., 2018; Chen, 2017; Chiu, 2012; Holroyd et al., 2011, 2013; Lin et al., 2007; Liu‐Chiang, 1993; Sun, 2015; Xu, 2020; Yeh et al., 2014). Furthermore, part of the impetus to modify such customs arose from the influence of Western health concepts and scientific notions, which undermined the credibility of some of those customs (Chen, 2017; Chiu, 2012; Liu‐Chiang, 1993; Xu, 2020; Yeh et al., 2014). In this regard, the postpartum women sought verification and support from online and professional sources in reshaping the ‘confinement’ practice to cope with the anticipated challenges (Chen, 2017; Xu, 2020).

#### 3.3.4 Theme 4: needs for family, social, and professional support in enhancing satisfaction with ‘confinement’

Eleven studies reported the postpartum women's needs for support to improve their ‘confinement’ experiences. Given their unfamiliarity with the complexities of the ‘confinement’ practice and with their new maternal role, the postpartum women (especially primiparas) wanted to have more information on how to better prepare themselves for confinement (Chen, 2017; Holroyd et al., 2013; Liu‐Chiang, 1993; Sun, 2015; Xu, 2020; Yeh et al., 2017). They wished to receive psychological support in the form of sympathy and understanding from their families, peers, and healthcare providers amidst their difficulties during ‘confinement’ (Chang et al., 2018; Chen, 2017; Chiu, 2012; Lin et al., 2007; Liu‐Chiang, 1993; Xu, 2020; Yeh et al., 2017; Zheng et al., 2019). Spousal support was deemed the most instrumental, as they hoped to share parenting duties with and obtain emotional support from their spouses (Chang et al., 2018; Chen, 2017; Chiu, 2012; Liu‐Chiang, 1993; Xu, 2020; Yeh et al., 2017). Peer support via access to other Chinese mothers was also valued to alleviate loneliness and isolation (Chang et al., 2018; Chen, 2017; Holroyd et al., 2013; Liu‐Chiang, 1993; Yeh et al., 2017).

For professional support, the postpartum women expressed their wish for informed and sensitive care from healthcare providers who understood the cultural practices and limitations the women faced during ‘confinement’ (Chang et al., 2018; Chen, 2017; Chiu, 2012; Yeh et al., 2017). In more practical terms, they would appreciate help from professional providers of postnatal care, with whose service they could enjoy greater control and autonomy, feel more comfortable, and avoid conflicts with family caregivers: ‘*I decided to leave it to the professional since they know what to eat to be beneficial, I can choose the menu myself … and it's delicious*’ (Chen, 2017; Chiu, 2012; Sun, 2015; Xu, 2020; Yeh et al., 2014).

## 4 DISCUSSION

This meta‐synthesis has integrated the experiences and needs of postpartum women undergoing the ‘confinement’ practice, through the elucidation of four themes, including their perception of it as an essential practice, physical and psychological stressors, coping strategies, and needs for support. The first theme in this review affirmed the postpartum women's perception of the significance of ‘confinement’, shaped by factors such as their socio‐cultural indoctrination and associations of the practice with health, as has been echoed in the literature (Liu et al., 2015; Vo & Desai, 2021). The interplay between these factors constituted powerful intrinsic motivators for the practice, accounting for their deep‐rooted perception of its significance and their continued adoption of its confinement customs. Finally, in clinical and practical settings, this theme highlighted the need for healthcare providers to recognize the importance of ‘confinement’ and incorporate these cultural considerations in caring for women following such customs.

The second theme in this review concerned the physical and psychological stressors during ‘confinement’, including burdensome behavioural restrictions, juggling multiple responsibilities, and intrusion from caregivers. Across most of the included studies, customary lifestyle prohibitions during ‘confinement’ were regarded by the postpartum women as taxing, inconvenient, and restrictive, with consequential stress and frustration. This finding has been corroborated by the literature, which underlined not only women's ambivalence and negativity towards such behavioural restrictions, but also the relationship between the restrictions and higher levels of depression (Qi et al., 2021; Yang et al., 2021). Given such negative sentiments, confinement practices should be re‐evaluated to identify modifiable or dispensable restrictions so that the postpartum women can enjoy greater convenience and freedom during ‘confinement’.

Juggling competing responsibilities of ‘confinement’ and motherhood has been demonstrated in this review to result in fatigue, anxiety, and stress, as has been mirrored in the literature (McCarthy et al., 2021; Rizzo & Watsford, 2020; Tsai & Wang, 2019). Compounding factors were also identified: difficulties in transitioning into motherhood (more prominent among primiparas), lack of confidence, and challenges in maternity (Tsai & Wang, 2019; Yeh et al., 2017). Across the studies, the postpartum women were observed to harbour socially constructed expectations of their roles and responsibilities in motherhood. Of note, the pressure to live up to these expectations has been reported to induce stress among them (McCarthy et al. (2021). Relevant to this context is the self‐discrepancy theory, which posits that, when reality does not match expectations, a discrepancy forms between an individual's ideal self and actual self, culminating in guilt, failure, and anxiety (Choi et al., 2005; Henderson et al., 2016; Higgins, 1987). Although this stressor might not relate exclusively to ‘confinement’ but rather generally to the postpartum experiences, this finding remained relevant in reflecting the ongoing stressors of confinement, as has been illustrated in several studies (Chang et al., 2018; Holroyd et al., 2004, 2011; Leung et al., 2005; Yeh et al., 2017; Zheng et al., 2019). Such stressors should be recognized by postnatal care providers in their provision of emotional affirmation, guidance, and support to postpartum women in fulfilling maternal responsibilities during ‘confinement’.

Interference from caregivers has represented another stressor. Conflicts over ‘confinement’ and approaches to infant care between the postpartum women and overzealous parents and mothers‐in‐law have often resulted in added stress, thereby offsetting benefits of family support and increasing chances of postpartum depression (Peng et al., 2021). This was commonly observed in Chinese societies, where care was intergenerational and shared with the extended family (Lin et al., 2021). As a result of the intergenerational pressure and unequal power relations in the traditional family unit, autonomy was eroded for postpartum women, who thus came under the control and authority of their elders at home, particularly their in‐laws (Lin et al., 2021; Wang et al., 2017). Likewise, prevalent patriarchal attitudes towards women's roles and responsibilities also undermined their autonomy in decision‐making (Peng et al., 2021). In view of these socio‐cultural dynamics, caregivers should considerately observe boundaries and ensure no encroachment upon both maternal and self‐autonomy of postpartum women under their care. Additionally, it merits attention that studies published in Chinese have provided more in‐depth discussions on such socio‐cultural dynamics than those in English, suggesting a gap regarding the impacts of cultural constructs on postpartum decisions in the literature.

Under the third theme of this review, various coping strategies (emotion‐ and problem‐based) used by postpartum women to mitigate the ‘confinement’‐related stressors were highlighted. Emotion‐based strategies involved mood regulation by relatively acquiescent postpartum women who opted to accommodate themselves to the ‘confinement’ customs. Conversely, problem‐based strategies involved modifying the burdensome customs and seeking professional support to avoid familial conflicts. A paradigm shift in the postpartum women's coping attitudes was observed across the studies: while older research emphasized acquiescence and acceptance (Lin et al., 2007; Liu‐Chiang, 1993), more recent studies highlighted liberation from customary restrictions through modified ‘confinement’ and professional support (Chang et al., 2018; Holroyd et al., 2011, 2013; Vo & Desai, 2021; Xu, 2020; Zheng et al., 2019). Several reasons underlay the shift in attitudes, including not only social fragmentation of traditional family units into nuclear ones, but also increasing education levels and scientific debunking of superstitions, both of which conferred postpartum women space and knowledge to modify the practices (Xu, 2020). Moreover, improved accessibility and availability of professional services further empowered them to exercise greater control and to prioritize their autonomy over their duty to traditions.

The postpartum women's needs for various support constituted the fourth theme in this review, including informational, psychological, and practical support from their family, peers, professionals, and online sources. All such identified support fell under social support, the need for which has likewise been reported in a meta‐analysis demonstrating its positive associations with lowered risks of postpartum depression (Qi et al., 2021). Additionally, the finding that spousal support was most important for the postpartum women has been paralleled in the literature, with a focus on the spouses' contributions in providing emotional support and childcare relief and in mitigating conflicts with the in‐laws (Tang et al., 2016; Wan et al., 2009). While most studies have emphasized spousal involvement (Chen, 2017; Shorey et al., 2021); support from male partners could not be guaranteed, given their traditionally entrenched roles in the Chinese culture as breadwinners, rather than as providers of domestic support. Thus, the need arose to educate postpartum women's spouses and family members on the importance of their roles beyond those dictated or stereotyped by their culture or traditions. This would be especially vital in providing the necessary support for positive confinement experiences, such as practical information on ‘confinement’ and motherhood, and emotional reassurance and validation. Lastly, where access to support from spouses and family was limited, support from peers and professional providers became crucial for postpartum women. The proliferation of online technologies and platforms can serve as viable alternative sanctuaries for them to seek informational and emotional support (Chang et al., 2018; Xu, 2020). Quality support during ‘confinement’ involved a trusting and empowering relationship with their care providers, who acknowledged the varying personal and cultural contexts in attending to their needs.

### 4.1 Strengths and limitations

Our research represented the first meta‐synthesis that pioneered the examination of qualitative studies published in English and Chinese databases exploring the experiences and needs of postpartum women undergoing ‘confinement’. It provided novel insights into the influences of various social and cultural constructs on the confinement experiences based on 13 studies from across five countries. Methodological rigour was maintained through the quality appraisal of the studies and the involvement of two reviewers who independently partook in the review to reduce biases and errors. The main limitation in this review was the lack of studies exploring ‘confinement’ among Chinese women residing in Western societies, which suggested a potential under‐representation of findings for this population and thus a gap for further research.

### 4.2 Implications to nursing and future research

Findings from this meta‐synthesis have emphasized postpartum women's determination to preserve the practice of ‘confinement’ arising from their established perception of its significance and explored the socio‐cultural milieu of their stressors, coping strategies, and needs for support. This review suggested a need to evaluate the ‘confinement’ practice and promote evidence‐based care to improve maternal well‐being. Nurses and midwives should be cognisant of such postpartum customs and adopt non‐judgemental attitudes when providing culturally sensitive care. As early as the prenatal period, nurses and midwives can pre‐emptively elicit beliefs and concerns on confinement among Chinese postpartum women under their care, to assess their readiness and support systems that the women may have. Having family‐focused strategies remain vital, given the central influence of the family unit on the confinement experiences among Chinese postpartum women. To minimize potential conflicts, it will be judicious for nurses and midwives to establish a shared understanding and expectations of ‘confinement’ with family members before the confinement. Given the increasing use of online platforms for support, the effectiveness of technological‐based interventions in delivering health education and information to support modifying ‘confinement’ can also be explored to provide additional insights on how to better support women partaking in this cultural phenomenon.

## 5 CONCLUSIONS

This review suggest that nurse will need to be aware and adopt non‐judgemental attitudes on postpartum customs and practices used by Chinese women when providing care as early as the prenatal period. Nurses will then be seen as providing culturally competent care to their client.

## AUTHORSHIP STATEMENT

*Study design*: Jocelyn Khor Yun Xin, Goh Yong Shian, and Wilson Tam Wai San. *Data collection*: Jocelyn Khor Yun Xin. *Data analysis*: Jocelyn Khor Yun Xin and Tan Ting Han. *Study supervision*: Wilson Tam Wai San and Goh Yong Shian. *Manuscript writing*: Jocelyn Khor Yun Xin, Goh Yong Shian, and Wilson Tam Wai San.

## CONFLICT OF INTEREST STATEMENT

No conflict of interest has been declared by the authors.

## ETHICS STATEMENT

Not applicable.

## Supporting information


**Table S1.** List of ‘Tso‐Yueh‐Tzu’ rules

**Table S2.** Search Strategy of Databases

**Table S3.** Data Extraction Form

**Table S4.** Quality appraisal of studies using CASP

**Table S5.** Derivation of Meta‐summarized Themes and Sub‐themes

**Table S6.** Derivation of Meta‐synthesized Themes

## Data Availability

Data sharing is not applicable to this article as no new data were created or analyzed in this study.
