# Supplementary material for: Experiences of postpartum Chinese women undergoing confinement practices: A qualitative meta‐synthesis
Source: Int J Nurs Pract. 2024 Feb 20;30(6):e13251. doi: 10.1111/ijn.13251 (PMC11608940; doi:10.1111/ijn.13251)
Supplement: Supplementary file 1 — Table S1. List of ‘Tso‐Yueh‐Tzu’ rules [file IJN-30-e13251-s003.docx]

# **Supplementary Table 1**

# *List of “Tso-Yueh-Tzu” rules*

| **Purpose** | **Diet** | **Behaviour** |
| --- | --- | --- |
| To restore yin-yang balance and regain energy | Eating ‘hot’ food to regain yin-yang balance, for example:   - Pigs trotters cooked with ginger and vinegar - Food cooked with rice wine - Chicken cooked in sesame oil - Shenghua tonic, a traditional tonic brewed from 10 herbs - Herbal soups with ginseng or other herbs - Pork liver and kidney - Red date, longan tea   Avoiding ‘cold’ food, for example:   - Fresh vegetables and fruits - Soybean products - Cold drinks - Ice cream   Avoid raw food and leftovers  Having more frequent meals | - Rest to ensure adequate energy - Sexual abstinence - Limiting visitors |
| To prevent illness |  | - To dress warmly - No washing of body or hair, especially avoiding contact with cold water. Only to soak body with ginger water - No brushing of teeth - Avoid wind, fans, and air conditioning. All windows sealed - Avoid walking or moving about; the ideal is lying on the back in bed - Avoid doing housework - Only to stay indoors, no outdoors - Do not carry heavy stuff or squat - Do not get sick - Do not read or cry |
| To increase breastmilk production | - Fish soup boiled in papaya - Avoid hot and spicy food |  |
| Perceived as “dirty,” inauspicious |  | - No going into another person’s home - No burning of incense or visit to a temple or altar |
