# Supplementary material for: Experiences of postpartum Chinese women undergoing confinement practices: A qualitative meta‐synthesis
Source: Int J Nurs Pract. 2024 Feb 20;30(6):e13251. doi: 10.1111/ijn.13251 (PMC11608940; doi:10.1111/ijn.13251)
Supplement: Supplementary file 2 — Table S2. Search Strategy of Databases [file IJN-30-e13251-s004.docx]

## Supplementary Table 2: Search Strategy of Databases

| **Database** | **Concept** | **Index terms and Keywords** | **Search Results (as of 20/12/21)** |
| --- | --- | --- | --- |
| **PubMed** | ‘Postpartum’  ‘Women’ | "Postpartum Period"[Mesh] OR Parturition[Mesh] OR postpartum[Title/Abstract] OR post-partum[Title/Abstract] OR postnatal[Title/Abstract] OR post-natal[Title/Abstract] OR postbirth[Title/Abstract] OR post-birth[Title/Abstract] OR child-birth[Title/Abstract] OR childbirth[Title/Abstract] OR parturition*[Title/Abstract] OR puerper*[Title/Abstract]  **AND**  Women[Mesh] OR Female[Mesh] OR Mothers[Mesh] OR women[Title/Abstract] OR woman[Title/Abstract] OR female*[Title/Abstract] OR mother*[Title/Abstract] OR matern*[Title/Abstract]  **AND** | **N=444** |
|  |  |  |  |
|  | ‘Doing-the-month’ | "Postnatal Care"[Mesh] OR “Social Support”[Mesh] OR peiyue[Title/Abstract] OR “pei yue”[Title/Abstract] OR tsoyueh*[Title/Abstract] OR "tso yueh"[Title/Abstract] OR zuoyue*[Title/Abstract] OR "zuo yue"[Title/Abstract] OR “doing the month”[Title/Abstract] OR "sitting month"[Title/Abstract] OR “sitting the month”[Title/Abstract] OR yuesao[Title/Abstract] OR "postnatal care"[Title/Abstract] OR "post-natal care"[Title/Abstract] OR "postpartum care"[Title/Abstract] OR "post-partum care"[Title/Abstract] OR "postpartum practice*"[Title/Abstract] OR "postnatal practice*"[Title/Abstract] OR "post-partum practice*"[Title/Abstract] OR "postpartum ritual*"[Title/Abstract] OR "post-partum ritual*"[Title/Abstract] OR "postpartum tradition*"[Title/Abstract] OR “postpartum custom*”[Title/Abstract] OR “postnatal custom*”[Title/Abstract] OR confinement[Title/Abstract] OR “social support”[Title/Abstract]  **AND** |  |
|  | ‘Chinese’ | "China"[Mesh] OR “Taiwan”[Mesh] OR "Singapore"[Mesh] OR "Malaysia"[Mesh] OR Chinese[Title/Abstract] OR China[Title/Abstract] OR Taiwan*[Title/Abstract] OR “Hong Kong”[Title/Abstract] OR Singapore*[Title/Abstract] OR Malaysia*[Title/Abstract] |  |
|  |  |  |  |
| **CINAHL** | ‘Postpartum’  ‘Women’ | MH ("Postnatal Period+") OR TI (postpartum OR “post-partum” OR postnatal OR post-natal OR post-birth OR “postbirth” OR childbirth OR parturition* OR puerper*) OR AB (postpartum OR “post-partum” OR postnatal OR post-natal OR post-birth OR “postbirth” OR childbirth OR parturition* OR puerper*)  **AND**  MH (women or female or mothers) OR TI (mother* OR matern* OR women OR woman OR female*) OR AB (mother* OR matern* OR women OR woman OR female*)  **AND** | **N = 430** |
|  | ‘Doing-the-month’ | (MH "Postnatal Care+") OR MH ("Support, Psychosocial") OR TI (peiyue OR “pei yue” OR tsoyueh* OR “tso-yueh” OR zuoyue* OR “zuo-yue” OR "doing-the-month” OR “doing-in-month” OR "sitting month” OR “sitting-the-month” OR yuesao OR (post-natal N3 care) OR (post-partum N3 care) OR (postpartum N3 care) OR (postnatal N3 care) OR (post-natal N3 ritual*) OR (post-partum N3 ritual*) OR (postpartum N3 ritual*) OR (postnatal N3 ritual*) OR (postpartum N3 practice*) OR (postnatal N3 practice*) OR (post-partum N3 practice*) OR (post-natal N3 practice*) OR (postpartum N3 tradition*) OR (postnatal N3 tradition*) OR (post-natal N3 tradition*) OR (post-partum N3 tradition*) OR (postpartum N3 custom*) OR (postnatal N3 custom*) OR (post-partum N3 custom*) OR (post-natal N3 custom*) OR confinement OR “social support”) OR AB (peiyue OR “pei yue” OR tsoyueh* OR “tso-yueh” OR zuoyue* OR “zuo-yue” OR "doing-the-month” OR “doing-in-month” OR "sitting month” OR “sitting-the-month” OR yuesao OR (post-natal N3 care) OR (post-partum N3 care) OR (postpartum N3 care) OR (postnatal N3 care) OR (post-natal N3 ritual*) OR (post-partum N3 ritual*) OR (postpartum N3 ritual*) OR (postnatal N3 ritual*) OR (postpartum N3 practice*) OR (postnatal N3 practice*) OR (post-partum N3 practice*) OR (post-natal N3 practice*) OR (postpartum N3 tradition*) OR (postnatal N3 tradition*) OR (post-natal N3 tradition*) OR (post-partum N3 tradition*) OR (postpartum N3 custom*) OR (postnatal N3 custom*) OR (post-partum N3 custom*) OR (post-natal N3 custom*) OR confinement OR “social support”)  **AND** |  |
|  | ‘Chinese’ | (MH "China+") OR (MH "Singapore") OR (MH "Malaysia") OR (MH "Taiwan") OR (MH “Hong Kong”) OR TI (Chinese OR China OR Taiwan* OR “Hong Kong” OR Singapore* OR Malaysia*) OR AB (Chinese OR China OR Taiwan* OR “Hong Kong” OR Singapore* OR Malaysia*) |  |
|  |  |  |  |
| **CNKI** | ‘Doing the month’ | TI= (“作月子” + “月子” + “做月子” + “坐月子” + “陪月” +“月嫂”) OR AB= (“作月子” + “月子” + “做月子” + “坐月子” + “陪月” +“月嫂” ) | **N = 1426** |
| **Airiti** | ‘Doing the month’ | ((((([ALL3]:(“作月子” ) OR [ALL3]:(“月子”)) OR [ALL3]:(“做月子” )) OR [ALL3]:(“坐月子” )))) | **N = 162** |
| **National Library of Theses and Dissertation (Taiwan)** | ‘Doing the month’ | "作月子".ti,ab,kw or "月子".ti,ab,kw or "坐月子".ti,ab,kw or "做月子".ti,ab,kw or “做月內”.ti,ab,kw or “陪月”.ti,ab,kw | **N = 241** |
