# Supplementary material for: Experiences of postpartum Chinese women undergoing confinement practices: A qualitative meta‐synthesis
Source: Int J Nurs Pract. 2024 Feb 20;30(6):e13251. doi: 10.1111/ijn.13251 (PMC11608940; doi:10.1111/ijn.13251)
Supplement: Supplementary file 3 — Table S3. Data Extraction Form [file IJN-30-e13251-s006.docx]

## Supplementary Table 3: Data Extraction Form

|  |  | **Remarks** |
| --- | --- | --- |
| Reviewer: |  |  |
| Primary study author(s): |  |  |
| Title of primary study: |  |  |
| Year of paper published: |  |  |

| **Study description** |  | **Remarks** |
| --- | --- | --- |
| Study Design |  |  |
| Method (recruitment): |  |  |
| Methodology/data collection method: |  |  |
| Setting (data collection): |  |  |
| Geographical (country/city): |  |  |
| Cultural (Language/ethnicity): |  |  |
| Participants (age/gender/relevant socio-demographics (e.g., education level): |  |  |
| Sample size: |  |  |
| Number of postpartum women: |  |  |
| Parity: |  |  |
| How long since postpartum? |  |  |
| Location of “Tso-Yueh-Tzu” |  |  |
| Caregiver during “Tso-Yueh-Tzu” |  |  |
| Data analysis method  (thematic analysis/content analysis/others): |  |  |
| Themes/  Sub-themes of primary study: |  |  |
| Authors conclusion: |  |  |
| Comments: |  |  |

| **Findings** | | |  |  |
| --- | --- | --- | --- | --- |
| Themes/subthemes | Verbatim (quotations): | Non-verbatim (author’s interpretations/words): | Page number | Remarks |
