# Supplementary material for: Experiences of postpartum Chinese women undergoing confinement practices: A qualitative meta‐synthesis
Source: Int J Nurs Pract. 2024 Feb 20;30(6):e13251. doi: 10.1111/ijn.13251 (PMC11608940; doi:10.1111/ijn.13251)
Supplement: Supplementary file 4 — Table S4. Quality appraisal of studies using CASP [file IJN-30-e13251-s002.docx]

# **Supplementary Table 4**

# *Quality appraisal of studies using CASP*

| **Authors and Year** | **Q1** | **Q2** | **Q3** | **Q4** | **Q5** | **Q6** | **Q7** | **Q8** | **Q9** | **Q10** | **Total Score** |
| --- | --- | --- | --- | --- | --- | --- | --- | --- | --- | --- | --- |
| Chang et al. (2018) | Y | Y | C | C | Y | Y | Y | Y | Y | Y | 8 |
| Leung et al. (2005) | Y | Y | C | Y | Y | N | Y | Y | Y | Y | 8 |
| Liu-Chiang (1993) | Y | Y | C | Y | Y | N | Y | Y | Y | Y | 8 |
| Chen (2017) | Y | Y | C | Y | C | N | C | N | Y | Y | 6 |
| Yeh et al. (2017) | Y | Y | C | Y | Y | Y | Y | N | Y | Y | 8 |
| Zheng et al. (2019) | Y | C | N | Y | C | N | Y | N | Y | Y | 6 |
| Yeh et al. (2014) | Y | Y | Y | Y | Y | Y | Y | N | Y | Y | 9 |
| Holroyd et al. (2013) | Y | Y | Y | Y | C | Y | Y | N | Y | C | 7 |
| Holroyd et al. (2011) | Y | Y | C | Y | Y | N | Y | N | Y | C | 6 |
| Sun (2015) | Y | Y | C | Y | Y | N | Y | Y | Y | Y | 8 |
| Chiu (2012) | Y | Y | Y | Y | Y | N | Y | Y | Y | Y | 9 |
| Lin et al. (2007) | Y | Y | C | Y | Y | Y | C | C | Y | C | 6 |
| Xu (2020) | Y | Y | C | Y | Y | N | C | N | N | C | 4 |

**Abbreviation: Y, Yes; N, No; C, Can’t tell**

Q1: Was there a clear statement of the aims of the research?

Q2: Is a qualitative methodology appropriate?

Q3: Was the research design appropriate to address the aims of the research?

Q4: Was the recruitment strategy appropriate to the aims of the research?

Q5: Was the data collected in a way that addressed the research issue?

Q6: Has the relationship between researcher and participants been adequately considered?

Q7: Have ethical issues been taken into consideration?

Q8: Was the data analysis sufficiently rigorous?

Q9: Is there a clear statement of findings?

Q10: How valuable is the research?
