# Supplementary material for: Experiences of postpartum Chinese women undergoing confinement practices: A qualitative meta‐synthesis
Source: Int J Nurs Pract. 2024 Feb 20;30(6):e13251. doi: 10.1111/ijn.13251 (PMC11608940; doi:10.1111/ijn.13251)
Supplement: Supplementary file 5 — Table S5. Derivation of Meta‐summarized Themes and Sub‐themes [file IJN-30-e13251-s001.docx]

## Supplementary Table 5: Derivation of Meta-summarised Themes and Sub-themes

| **Concepts from the included studies** | **Meta-summarised results** | |
| --- | --- | --- |
|  | **Sub-themes** | **Themes** |
| Zuoyuezi is essential | Zuoyuezi is essential | Zuoyuezi being essential (effect size: 92.3%) |
| Old tradition having its reasons |  |  |
| Postpartum women perceive postpartum period as being in a vulnerable state | Being vulnerable and weak during postpartum |  |
| Feeling weak after childbirth |  |  |
| Care of self as the central belief of postpartum women |  |  |
| Association of confinement practices and health | Belief in health effects of zuoyuezi |  |
| Believing in the negative health impacts from not following “zuoyuezi.” |  |  |
| Believing in the positive health impacts from following zuoyuezi |  |  |
| Concerned about possible negative health consequences from failing to follow all of the elements of “zuo yue zi” |  |  |
| Women believe that doing zuoyuezi can help them recover |  |  |
| Concerns about not sleeping well during during zuoyuezi | Wanting to achieve adequate rest | Need for adequate rest (effect size: 92.3%) |
| Emphasis on getting enough rest during zuoyuezi to recuperate to regain energy to look after baby |  |  |
| Postpartum discomfort would subside with proper rest |  |  |
| Feeling exhausted and physically uncomfortable | Feeling exhausted |  |
| Lack of sleep and adequate rest during confinement from juggling infant care responsibilities simultaneously |  |  |
| Zuoyuezi rooted in Chinese culture | Cultural pressure | Sociocultural pressure to undergo zuoyuezi (effect size: 92.3%) |
| Doing-the-month being extremely significant for Chinese women |  |  |
| Being immersed in cultures where everyone was following the principles of “zuo yue zi” |  |  |
| Pressure and influence from older generation to undergo zuoyuezi | Older generation’s influence |  |
| Compelled to defer to elders instead of own principles |  |  |
| “There is no harm” in elders’ advice |  |  |
| “Everyone does it like this” | Societal pressure |  |
| Expectations to zuoyuezi reinforced by families and friends, online and books |  |  |
| Influenced by personal experiences of others who have done the month |  |  |
| Modification of zuoyuezi | Modification of zuoyuezi practices | Modification of zuoyuezi practices (effect size: 76.9%) |
| Seeking trusted sources of knowledge to guide zuoyuezi adjustments |  |  |
| Choosing a hybrid confinement model combining both traditional and modern concepts |  |  |
| Burdensome nature and efficacy of certain practices | Burdensome |  |
| Difficulties in adhering to traditional proscriptions: resulted in dizziness and constipation | Discomfort |  |
| Behavioural taboos causing inconvenience or discomfort, especially no washing of hair and diet |  |  |
| Value of science and comfort in doing-the-month | Pursuit of contemporary scientific knowledge |  |
| Adjustment and fine-tuning of traditional rituals based on contemporary scientific knowledge and logic |  |  |
| Traditional approach regarded outdated due to advancements in knowledge and science |  |  |
| More inclined to think independently and use modern scientific approach in confinement over traditional methods |  |  |
| Questioning relevance and significance of some zuoyuezi practices | Doubt surrounding zuoyuezi practices |  |
| Verifying the meaning and rules of tso-yueh-tzu practices from several sources |  |  |
| Questioning health impact of some zuoyuezi dietary proscriptions |  |  |
| Perceiving certain traditional customs as not making sense |  |  |
| Disagreement with traditional taboos and ancient way of confinement |  |  |
| Influence of American postpartum norms on deflecting own cultural postpartum practices | Cross-cultural influence |  |
| Availability and accessibility of platforms to gain support and information needs from other women with postpartum experience | Availability and accessibility of supporting resources | The need for support from others (effect size: 92.3%) |
| Preference for paid help over family’s help when conflict arises | Turning to professional postpartum help |  |
| Preference for PNCs over mother-in-law’s care as they felt uncomfortable with mother-in-law |  |  |
| Preference for PNCs due to expertise knowledge in maternal and infant care |  |  |
| Commercialised options afford women sense of control, autonomy and emotional well-being |  |  |
| Doing the month at PNCs enable them to be self-focused, increase opportunities to rest, receive proper nutrition, and restore their energy |  |  |
| Being at PNC is more comfortable and involves fewer restrictions |  |  |
| Desperate for help in doing-the-month | Practical support |  |
| Help and support from care providers in household chores and infant care provided postpartum women with sufficient time to rest and recuperate |  |  |
| Women place value in spouse’s involvement over care of infant | Spousal support |  |
| Preference for supportive healthcare providers, who were aware of and sensitive to their cultural practices | Support from healthcare professionals |  |
| Appreciation for knowledgeable nurses on zuoyuezi practices |  |  |
| Healthcare professionals’ lack of awareness of zuoyuezi practices |  |  |
| Healthcare professionals lack of respect of women’s beliefs about zuoyuezi |  |  |
| Peer visit made things better for postpartum women | Emotional support |  |
| Support from friends alleviating feelings of isolation |  |  |
| Interacting with other mothers helped alleviate loneliness |  |  |
| Relationships with supportive Chinese family members and friends catalysed their experiences of “zuo yue zi” |  |  |
| Emotional connection being the most valuable thing over physical separation |  |  |
| Psychological support from families provided women warmth and connection |  |  |
| Making preparation for zuoyuezi and looking for information | Information support |  |
| Unfamiliar with complex doing the month procedures |  |  |
| The need to gain more information on caregiving skills |  |  |
| Sources of information on zuoyuezi came from Chinese family members, friends, online information and books |  |  |
| Postpartum women felt childcare support was insufficient | Inadequacies in support |  |
| Lack of guidance and support from midwives and staff |  |  |
| Lack of psychological support in adjustment to maternal role |  |  |
| Reduction of social support resulted in poor maternal sleep quality, creating or exacerbating postpartum depression |  |  |
| Lack of attention and support from husband in looking after them and baby |  |  |
| Feeling happy during doing-the-month from receiving adequate support and encouragement | Feeling satisfied | Satisfaction towards zuoyuezi (effect size: 38.5%) |
| Everything went smoothly with help of paid professional helpers |  |  |
| Happy to follow traditional diet practices to promote recovery |  |  |
| Feeling unhappy and crying sometimes | Low mood | Distress during zuoyuezi (effect size: 84.6%) |
| In a foul mood often during zuoyuezi |  |  |
| Feeling uncomfortable doing-the-month at in-law’s | Stress and frustration |  |
| Wouldn’t feel at ease doing the month at in-law |  |  |
| Feeling stressed doing-the-month with in-law |  |  |
| Feeling stressed about postpartum rituals contrasting personal hygiene practices |  |  |
| Frustration due to differing opinions in postpartum care |  |  |
| Frustration due to culturally insensitiveness from healthcare professionals on zuoyuezi practices |  |  |
| Emotional frustration due to difficulties in adjusting to the confinement taboos and grappling with role changes into motherhood |  |  |
| Feeling trapped and guarded from not being able to go out | “Being in prison” |  |
| “Being in prison” |  |  |
| Lack of autonomy or breathing space when doing-the-month with mothers-in-law |  |  |
| Feeling dejected and suffocated, and like being in jail from lack of freedom from confinement taboos |  |  |
| Restricted freedom and mobility while doing confinement at home | Feeling restricted |  |
| Troubled by loss of privacy and inconvenience of doing-the-month with in-law | Loss of privacy and personal space |  |
| “Had no choice but to endure the taboos” | “No choice but to endure” | Putting up with confinement taboos (effect size: 92.3%) |
| Persisting with confinement norms despite discomfort | Tolerating as much as possible |  |
| Tolerate it as much as I can |  |  |
| Trying their best to abide by the traditional ways of confinement as much as possible |  |  |
| Not in control of childcare and postpartum decisions due to maintenance of intergenerational hierarchy | Losing control and autonomy | Control and decision-making power (effect size (69.2%) |
| Pressure of discipline and external surveillance during the period of “doing-the-month”, which made them feel under control and isolation during the month |  |  |
| Lack of decision-making power and having to conform to family elders authority and control over postpartum and infant care decision | Submitting to traditional family authority and no say in decision-making |  |
| Lack of power to speak at home from being in a relatively weaker position |  |  |
| Dared not verbalise disagreement or express how they felt about confinement taboos |  |  |
| Commercialised options afford women sense of control, autonomy and emotional well-being | Gaining control and autonomy |  |
| The ability to make their own decisions and doing it my way |  |  |
| “When it comes to childcare, my opinions matter the most” |  |  |
| Challenging traditional authoritative voices of older elders |  |  |
| Poor relationship with postpartum helpers made them unhappy | Relationship with external postpartum helpers | Maintaining relationships with care helpers (effect size: 100%) |
| Doubt in postpartum doula’s credentials and knowledge in maternal and infant care |  |  |
| Being satisfied with zuoyuezi and postpartum helpers |  |  |
| Feeling closer with spouse with sharing of infant care responsibility | Spousal relationship |  |
| Husband’s lack of empathy and involvement during zuoyuezi |  |  |
| Dissatisfaction with lack of attention and support from husband in looking after them and the baby |  |  |
| Strained relationship with mother-in-law | Strain in relationship with mother-in-law |  |
| Mother-in-law’s help perceived unwelcome at times, since women found it difficult to express disagreement with their mother-in-law |  |  |
| Reluctance and fear of speaking up to express their opinions to their mother-in-law |  |  |
| Difficulties in communication with mother-in-law over postpartum and infant care |  |  |
| Friction and conflict with family members over differing zuoyuezi expectations and infant care | Tension and conflict over differing expectations |  |
| Constraint and emotional stress due to friction between filial obligations and intergenerational differences |  |  |
| Modifying mood and thinking positively to manage conflicts | Conflict management |  |
| Finding ways to cope with confinement taboos secretly without mother-in-law’s knowing |  |  |
| Reaching for consensus with caregivers on zuoyuezi norms and infant care practices |  |  |
| Negotiating generational differences in zuoyuezi practices |  |  |
| Forced to give in and compromise to avoid conflict and maintain family harmony |  |  |
| Turning to husbands to help negotiate conflicts with mother-in-law |  |  |
| Appreciation and recognition of support for family elders helped to maintain a good relationship |  |  |
| Cost for professional zuoyuezi services being quite burdensome | Financial concerns | Financial constraints (effect size: 46.2%) |
| Lack of financial ability hindered postpartum women from choosing their preferred confinement method |  |  |
| Ending confinement early to return to the workplace | Work pressure |  |
| Expectation to maintain family harmony by respecting the elders’ wishes | Expectations of others | Navigating expectations (effect size: 84.6%) |
| Care providers demanded strict adherence to zuoyuezi ritual |  |  |
| Expecting to receive care from others for themselves and their baby | Self-expectations |  |
| Concerns over whether paid help met their zuoyuezi expectations |  |  |
| Utilize the period of confinement to learn about newborn care and so facilitate their transition to motherhood | Internalise mothering | Juggling motherhood duties (effect size: 92.3%) |
| Wanting to step up on in infant care duties, as part of mother’s responsibility |  |  |
| Feeling overwhelmed and helpless at times when the baby cries | Uncertainty and anxiety regarding infant care |  |
| Feeling uncertain with the sudden arrival of the child and juggling with adjusting to embrace role change into a mother |  |  |
| Trepidation and confusion regarding infant care in first month postpartum |  |  |
| Concern about unending and overwhelming childcare duties |  |  |
| Feeling incapable and incompetent in child care responsibilities | Lacking confidence |  |
| Not sure if I will be a ‘good mother’ |  |  |
| Unable to maintain adequate contact in mother-infant bonding | Challenges in motherhood duties |  |
| Lack of necessary skills and knowledge in infant care |  |  |
| Sleep disruption from struggling with infant care at night |  |  |
| Stress and discomfort brought on by breast engorgement and postpartum pain |  |  |
| Feeling tired from juggling breastfeeding during Tso-Yueh-Tzu |  |  |
| Worried about postpartum physical changes to their body, especially their outer appearance | Concerns about body image and figure | Maintenance of self (effect size: 42.9%) |
| Worried about restoration of body figure |  |  |
| Worries about postpartum food being fattening |  |  |
| Anxiety about going back to work without resuming pre-pregnancy image |  |  |
| Struggle to maintain consistent self and body appearance | Preserving sense of self |  |
| Not wanting to lose themselves in the many rules dictated by the ritual |  |  |
| Determination to preserve one’s self determination during zuoyuezi |  |  |
| Care of self being the central belief of postpartum women |  |  |

| **Meta-summarised themes** | **Corresponding studies** |
| --- | --- |
| Zuoyuezi being essential (n=12) | Chang et al. (2018); Chen (2017); Chiu (2012); Holroyd et al. (2011); Holroyd et al. (2013); Leung et al. (2005); Lin et al. (2007); Sun (2015); Xu (2020); Yeh et al. (2014); Yeh et al. (2017); Zheng et al. (2019) |
| Need for adequate rest (n=12) | Chang et al. (2018); Chen (2017); Chiu (2012); Holroyd et al. (2011); Holroyd et al. (2013); Leung et al. (2005); Lin et al. (2007); Sun (2015); Xu (2020); Yeh et al. (2014); Yeh et al. (2017); Zheng et al. (2019) |
| Sociocultural pressure to undergo zuoyuezi (n=12) | Chang et al. (2018); Chen (2017); Chiu (2012); Holroyd et al. (2011); Holroyd et al. (2013); Leung et al. (2005); Lin et al. (2007); Liu-Chiang (1993); Sun (2015); Xu (2020); Yeh et al. (2014); Zheng et al. (2019) |
| Modification of zuoyuezi practices (n=10) | Chang et al. (2018); Chen (2017); Chiu (2012); Holroyd et al. (2011); Holroyd et al. (2013); Lin et al. (2007); Liu-Chiang (1993); Sun (2015); Xu (2020); Yeh et al. (2014) |
| The need for support from others (n=12) | Chang et al. (2018); Chen (2017); Chiu (2012); Holroyd et al. (2013); Leung et al. (2005); Lin et al. (2007); Liu-Chiang (1993); Sun (2015); Xu (2020); Yeh et al. (2014); Yeh et al. (2017); Zheng et al. (2019) |
| Satisfaction towards zuoyuezi (n=5) | Chang et al. (2018); Chen (2017); Chiu (2012); Lin et al. (2007); Zheng et al. (2019) |
| Distress during zuoyuezi (n=11) | Chen (2017); Chiu (2012); Leung et al. (2005); Liu-Chiang (1993); Holroyd et al. (2011); Holroyd et al. (2013); Lin et al. (2007); Sun (2015); Xu (2020); Yeh et al. (2017); Zheng et al. (2019) |
| Putting up with confinement taboos (n=12) | Chen (2017); Chiu (2012); Holroyd et al. (2011); Holroyd et al. (2013); Lin et al. (2007); Liu-Chiang (1993); Sun (2015); Xu (2020); Yeh et al. (2014); Yeh et al. (2017); Zheng et al. (2019) |
| Control and decision-making power (n=9) | Chen (2017); Chiu (2012); Holroyd et al. (2011); Lin et al. (2007); Leung et al. (2005); Liu-Chiang (1993); Sun (2015); Xu (2020); Yeh et al. (2014) |
| Maintaining relationships with care helpers (n=13) | Chang et al. (2018); Chen (2017); Chiu (2012); Holroyd et al. (2011); Holroyd et al. (2013); Leung et al. (2005); Lin et al. (2007); Liu-Chiang (1993); Sun (2015); Xu (2020); Yeh et al. (2014); Yeh et al. (2017); Zheng et al. (2019) |
| Financial constraints (n=6) | Chang et al. (2018); Chen (2017); Chiu (2012); Liu-Chiang (1993); Sun (2015); Xu (2020) |
| Navigating expectations (n=11) | Chang et al. (2018); Chen (2017); Chiu (2012); Holroyd et al. (2011); Leung et al. (2005); Lin et al. (2007); Liu-Chiang (1993); Sun (2015); Xu (2020); Yeh et al. (2014); Zheng et al. (2019) |
| Juggling motherhood duties (n=12) | Chang et al. (2018); Chen (2017); Chiu (2012); Holroyd et al. (2013); Leung et al. (2005); Liu-Chiang (1993); Sun (2015); Xu (2020); Yeh et al. (2014); Yeh et al. (2017); Zheng et al. (2019) |
| Maintenance of self (n=6) | Chen (2017); Chiu (2012); Liu-Chiang (1993); Sun (2015); Xu (2020); Yeh et al. (2014) |
