# Supplementary material for: Experiences of postpartum Chinese women undergoing confinement practices: A qualitative meta‐synthesis
Source: Int J Nurs Pract. 2024 Feb 20;30(6):e13251. doi: 10.1111/ijn.13251 (PMC11608940; doi:10.1111/ijn.13251)
Supplement: Supplementary file 6 — Table S6. Derivation of Meta‐synthesized Themes [file IJN-30-e13251-s005.docx]

## Supplementary Table 6: Derivation of Meta-synthesised Themes

| **Meta-summarised results** | | **Meta-synthesised results** |
| --- | --- | --- |
| **Themes** | **Subthemes** | **Themes and subthemes** |
| Sociocultural pressure to undergo zuoyuezi | Cultural pressure | 1a. “Tso-Yueh-Tzu” as an essential practice *(Deep-rooted social indoctrination)* |
|  | Older generation’s influence |  |
|  | Societal pressure |  |
| Zuoyuezi is essential | Holding onto tradition despite Western influence |  |
|  | Being vulnerable and weak during postpartum | 1b. “Tso-Yueh-Tzu” as an essential practice *(Health association)* |
|  | Belief in health effects of zuoyuezi |  |
|  | Personal experience |  |
| Need for adequate rest | Wanting to achieve adequate rest |  |
| Modification of zuoyuezi practices | Burdensome nature | 2a. Physical and psychological stressors of “Tso-Yueh-Tzu” *(Burdensome rituals)* |
| Need for adequate rest | Feeling exhausted |  |
| Modification of zuoyuezi practices | Discomfort |  |
| Distress during zuoyuezi | Stress and frustration |  |
|  | Low mood |  |
|  | “Being in prison” and being restricted |  |
| Financial constraints | Financial concerns |  |
| Juggling motherhood duties | Uncertainty and anxiety regarding infant care | 2b. Physical and psychological stressors of “Tso-Yueh-Tzu” (*Juggling multiple responsibilities)* |
|  | Lacking confidence |  |
|  | Challenges in motherhood duties |  |
|  | Internalise mothering |  |
| The need for support from others | Inadequacies in support |  |
| Relationships with care helpers | Tension and conflict over differing expectations | 2c. Physical and psychological stressors of “Tso-Yueh-Tzu” *(Negative interference)* |
|  | Strain in relationship with mother-in-law |  |
| Distress during zuoyuezi | Stress and frustration |  |
|  | Troubled by loss of privacy and personal space |  |
| Control and decision-making power | Loss of control and autonomy |  |
| Maintaining relationships with care helpers | Relationship with external postpartum helpers |  |
| Control and decision-making power | Submitting to traditional family authority | 3a. Coping strategies postpartum women used (*Giving in and maintaining tolerance)* |
| Navigating expectations | Expectations of others to conform |  |
| Putting up with confinement taboos | “No choice but to endure” |  |
|  | Tolerating as much as possible |  |
| Managing relationships with care helpers | Conflict management |  |
| Modification of zuoyuezi practices | Pursuit of contemporary scientific knowledge | 3b. Coping strategies postpartum women used (*Reshaping tradition to preserve self-determination)* |
|  | Doubt surrounding zuoyuezi practices |  |
| Modification of zuoyuezi practices | Discomfort |  |
| Maintenance of self | Maintaining body image and figure |  |
|  | Preserving sense of self |  |
| The need for support from others | Inadequacies in support | 4. Family, social and professional support needs in enhancing satisfaction of “doing-the-month” |
|  | Information support |  |
|  | Emotional support from families and friends |  |
|  | Informed and sensitive care from HCPs |  |
|  | Spousal support |  |
|  | Practical support in household chores and infant care |  |
|  | Turning to professional postpartum support |  |
| Control and decision-making power | Wanting to gain control and autonomy |  |
| Maintaining relationships with care helpers | Spousal Relationship |  |
| Satisfaction towards zuoyuezi | Feeling satisfied |  |
|  | Appreciation |  |
